# Gunshot Wounds of the Small Intestines

**Published:** 1884-09

**Authors:** 


GUN-SHOT WOUNDS OF THE SMALL INTESTINES. By Charles T. Parkes, M. D., Professor of Anatomy in Rush Medical College, Chicago, Ill. Being the address of the Chairman of the Section on Surgery and Anatomy read at the meeting of the American Medical Association held in Washington, D. C., May, 1884.

This essay is issued in pamphlet form, with appropriate illustrations by the author, to whom we are under obligations for a copy; and if the favorable results of his pains-taking experiments on dogs can be secured in accidents to the human race, this contribution should serve to set aside the hue and cry from certain quarters against vivisections in inferior animals. When it is understood that anesthetics were used "previous to being hurt" no charge of cruelty to animals will hold in these experiments.

The gynecologists will doubtless criticise the want of care in the two "deaths that occurred from secondary hemorrhage from uterine stumps—the ligature having slipped."

It is to be regretted that in the case which called for resection of the ileo cœcal valve, the animal soon died, as this experiment has much practical importance in view of the proneness of this portion of the alimentary tube to disease and also to intussusception.

The record of cases is interesting and instructive, so that it will repay a careful perusal.

